# Differential Expressions of Immunoregulatory microRNAs in Breast Milk of Mothers of Preterm Versus Term Infants: A Preliminary Study

**DOI:** 10.3390/medicina61091560

**Published:** 2025-08-29

**Authors:** Claudio Villota Arcos, Emanuel Jeldes Jerez, Jorge Carrasco Contreras, Mauricio Bittner Ortega, Susana Contreras Duarte, Ángel Roco Videla

**Affiliations:** 1 Escuela de Nutrición y Dietética, Facultad de Ciencias de la Salud, Universidad Bernardo O’Higgins, Santiago 8370993, Chile; claudio.villota@ubo.cl (C.V.A.); carrasco.jorge@pregrado.ubo.cl (J.C.C.); 2 Escuela de Medicina, Facultad de Ciencias Médicas, Universidad Bernardo O’Higgins, Santiago 8370993, Chile; 3 Centro Científico y Tecnológico de Excelencia Ciencia y Vida, Santiago 8340148, Chile; e.jeldes@ed.ac.uk; 4 Centre for Inflammation Research, Institute for Regeneration and Repair, University of Edinburgh, Edinburgh EH166 4UU, UK; 5 Laboratorio de Microbiología y Biotecnología Oral, Facultad de Ciencias de la Vida, Universidad Andrés Bello, Santiago 8370035, Chile; mbittner@unab.cl; 6 School of Nutrition and Dietetics, Faculty of Rehabilitation and Quality of Life Sciences, Universidad San Sebastián, Concepción 4080871, Chile; susana.contreras@uss.cl; 7 Vicerrectoría de Investigación e Innovación, Universidad Arturo Prat, Iquique 1110939, Chile

**Keywords:** microRNAs, human breast milk, term and preterm infants, immune response

## Abstract

*Background and Objectives*: Human breast milk contains essential nutrients for infant growth, as well as bioactive molecules such as exosomes and miRNAs, which play a key role in the maturation of the infant’s immune system. Breast milk from mothers of preterm and term infants shows significant differences in its nutrient contents and bioactive components. This preliminary study aimed to compare the expressions of 13 immunomodulatory microRNAs present in breast milk from the mothers of preterm and term infants. *Materials and Methods*: Breast milk samples were obtained from 5 breastfeeding mothers of term infants and 5 breastfeeding mothers of preterm infants. Every mother provided morning, noon, and evening milk samples. The total protein, fat, and lactose concentrations were measured. In addition, miRNAs were extracted from the exosomal fraction of each sample. The expression levels of the 13 miRNAs were compared and analyzed at the three time points each day. *Results*: The preterm infants’ milk had higher average fat and lactose levels. There were no differences in the total protein concentrations. The expressions of miRNAs in the preterm infants’ milk showed significantly higher variations in miR-17-5p, miR-24, miR-29b, miR-30a-5p, and miR-146a. The other miRNAs did not show variations. Interestingly, the highest miRNA expression was only observed in breast milk from the nighttime. The morning and midday samples showed distinct expression patterns. *Conclusions*: We identified the immunomodulatory microRNA components and their changes in expression levels at different times of the day, as well as those most strongly expressed in breast milk consumed by preterm infants.

## 1. Introduction

Breast milk plays a fundamental role in infant development, providing essential nutrients to the newborn, such as proteins, lipids, essential carbohydrates, vitamins, and minerals [1]. Breast milk can promote the development of the infant’s brain, gut, and immune system, among other benefits [2]. In addition to nutrients, breast milk provides many bioactive components, such as antibodies and RNAs, that modulate the infant’s immune response [3]. Most of these bioactive components are contained in extracellular vesicles, which vary in size and content, depending on their origin [4]. The most studied extracellular vesicles are known as exosomes, ranging in size from 30 to 150 nm [5]. Extracellular milk vesicles are produced and released by the luminal epithelium, resisting digestion and being absorbed by recipient cells in the infant [6,7,8,9,10]. Extracellular breast milk vesicles modulate the expressions of target genes [6] and regulate metabolic processes [9] and the infant’s immune response [11,12,13]. One of the main bioactive components of extracellular vesicles is microRNAs [14]. MicroRNAs, or miRNAs, are small non-coding RNAs of approximately 22 nucleotides in length [14]. These miRNAs regulate gene expression through the repression or degradation of messenger RNAs [15].

Currently, many miRNAs have been identified in both human and cow’s milk. The abundance and diversity of these miRNAs in breast milk have been linked to certain maternal diseases [16]. Furthermore, it has been shown that during lactation, the expression profiles of these transcripts change until the production of mature breast milk [17]. Interestingly, miRNA expression profiles, like those of nutrients such as proteins and fats, also change under time feeding conditions [18].

When comparing the contents of human milk (HM) consumed by preterm and term infants, significant differences have been observed in the levels of fats, proteins, antibodies, and cells, among others [19,20,21].

Similar results have been reported when comparing the miRNA expression levels in milk from the mothers of term and preterm infants [22,23]. At least 30 miRNAs have been identified in breast milk, and their levels correlate with the infant’s maturity [24]. However, few reports have evaluated the expressions of microRNAs regulated by nutrients such as vitamins or minerals. The microRNAs analyzed are related to inflammation, the innate or acquired immune response, immunotolerance, or cytotoxic effects. Briefly, miR16-5p and miR146a reduce the macrophage-mediated pro-inflammatory responses, such as TNF-α, IL-6, and IFN-β [21]. miR16-5p downregulates the expression of programmed cell death-ligand 1(PD-L1), a pivotal immune suppressor that controls macrophage–T-cell interaction and T-cell activation. miR16-5p induces macrophage polarization from M_2_ to M_1_ and activates CD4 + T cells by downregulating PD-L1 [21]. miR191-5p increases the survival of T cells throughout STAT5 phosphorylation mediated by JAK1 and JAK3 [25]. miR-55-5p regulates the interferon response, macrophage polarization, and the activity of NK cells [26].

miR223-5p attenuates M_1_ macrophage polarization by suppressing the Notch signaling pathway and Nod-like receptor protein 3 (NLRP3)-mediated pathway [27]. miR-223 is also involved in the regulation of the antigen presentation ability. miR-24 attenuates the stimulator of interferon gene (STING) pathway [28]. let-7a and let-7b miRNAs bind to Toll-like receptor 7 (TLR7) and TLR8 (receptors involved in the innate immune response) [29,30]. miR30a suppresses TLR/MyD88 activation and the cytokine expression in THP-1 cells [31]. miR-223-5p directly targets TLR2, TLR3, or TLR4. miR150-5p regulates the maturation of B cells by the downregulation of the transcriptional factor c-Myb [32]

The present study aimed to determine, in a pilot trial, the expression levels of 13 miRNAs in human breast milk from mothers of preterm and term infants. Each mother took three samples: one from the morning, one from the afternoon (noon), and one from the evening (nighttime). The miRNAs analyzed have been previously described as regulators of the immune response.

## 2. Materials and Methods

### 2.1. Milk Sample Collection

Milk samples were collected from 5 healthy mothers of preterm infants and 5 healthy mothers of term infants (Table 1) during the first 48 h after delivery and at 30 days postpartum. The inclusion criteria were as follows: (a) mothers of infants between 2 days and 1 month old; (b) mothers of infants living in the Metropolitan Region of Santiago, Chile; (c) mothers whose infants are growing appropriately for their age; (d) healthy mothers and infants. The exclusion criteria were as follows: (a) mothers who smoke or who use alcohol and/or drugs; (b) mothers of infants with nutritional problems or whose growth is inappropriate for the infant’s age; (c) mothers of twin infants. Participants manually extracted milk samples into 50 mL sterilized, polypropylene collection tubes (Corning, Life Sciences, Tewksbury, MA 01876, USA). Each mother took three samples: one from the morning, one from the afternoon (noon), and one from the evening (nighttime). Each sample was processed separately. The samples were transported to the laboratory in containers at 4 °C and stored at −80 °C pending further analysis.

### 2.2. Breast Milk Macronutrient Determination

One milliliter of breast milk samples was heated in a thermoregulating water bath at 40 °C and vortexed for 10 s to ensure biofluid homogeneity. The percentages (%) of fat, protein, and lactose in whole breast milk were measured using Foss MilkoScan FT-plus (Foss Electric A/S, Hillerød, Denmark).

### 2.3. Milk Fractionation

The milk samples were fractionated by centrifugation at 6500× *g* for 30 min at 4 °C. Two fractions were obtained from each sample: the fat and skim milk. The skim milk and the lipid layer were transferred separately to different tubes. The skim milk was centrifuged at 12,000× *g* for 1 h at 4 °C to remove debris. The defatted supernatant was then passed through 5 μm and 0.45 μm filters to remove residual debris.

### 2.4. Exosome Extraction

For the exosome extraction, 2 mL of eluate milk was mixed with 500 μL of ExoQuick exosome precipitation solution (EXOQ20A-1, System Biosciences, Palo Alto, CA 94303, USA) and incubated for 12 h at 4 °C. The resulting mixture was centrifuged for 30 min at 1500× *g* to obtain the fraction of exosomes in the pellet. The concentration and quality of the exosomes, along with their size, were determined using a NanoSight NS300 (Malvern Panalytical, 7602 EA Almelo, The Netherlands). The purified exosomes were stored at −80 °C until used.

### 2.5. Exosome Quantification and Size Profiling

The number of exosomes was determined using the Exocet kit (System Biosciences, Palo Alto, CA 94303, USA), according to the manufacturer’s instructions. The exosomes were lysed using a gentle lysis solution to preserve the enzymatic activity of the exosomal Acetylcholinesterase (AChE) enzyme. A standard curve was generated using known numbers of exosomes measured by NanoSight. The number and size of the exosomes were determined by nanoparticle tracking analysis (NTA) equipped with a 532 nm laser (NanoSight LM10 system, Malvern Panalytical, 7602 EA Almelo, The Netherlands). The vesicle size distribution and an approximate concentration were obtained from the raw data displayed by the associated software. The experiments were conducted in triplicate.

### 2.6. Extraction of microRNAs

The total RNAs from breast milk exosomes were extracted using the mirVana miRNA Kit (Ambion Life Technologies, Austin, TX, 78744, USA), following the manufacturer’s instructions. Briefly, the exosomes were diluted in two volumes of lysis solution and mixed vigorously for 30 s, and they were then incubated for 5 min at room temperature. Subsequently, a 1/10 volume of the additive was added, mixed vigorously for 30 s, and incubated for 10 min on ice. An equal volume of phenol/chloroform (Ambion Life Technologies, Austin, TX, 78744, USA) was added to each product and mixed vigorously, stirred for 1 min, and then centrifuged for 10 min at 10,000× *g*. The resulting aqueous phase was vigorously mixed with 1.25 volumes of 100% Ethanol and filtered through the mirVana column in sequences of 700 μL each time. The column was washed with mirVana wash solution, and the RNA was eluted using nuclease-free water at 95 °C. The concentration of RNA was determined by means of analysis on the Epoch™ Spectrophotometer System (Agilent, Santa Clara, CA 95051, USA). The extracted RNA was stored at −80 °C.

### 2.7. Amplification of microRNAs

For the RNA samples, 200 ng of RNA was used to prepare cDNA using the miScript II RT Kit system (Qiagen, Hilden, Germany). After cDNA synthesis, an equivalent of 1 ng of the original RNA sample was mixed with the miScript SYBR Green system (Qiagen, Hilden, Germany) using the reverse universal splitter included in the miScript II RT Kit system (Qiagen, Hilden, Germany). The following pre-designed microRNA assay primers were used in 15 μL qPCR reactions: miR-17-5p: CAAAGTGCTTACAGTGCAGGTAG; miR-24: CGCACATGACTCGTAGATACGG; miR-29b: GCAAGCCCTAGTATTCCTCGAC; miR-30a-5p: AATGCTTGCTCGGTATTAGCCT; miR-146a: TAACCGAATCTTGCCATACGCA; miR-150-5p: TCTCCCAACCCTTGTACCAGTG; miR155-5p: TTAATGCTAATCGTGATAGGGGTT; miR-16-5p: TAGCAGCACGTAAATATTGGCG; miR-191-5p: CAACGGAATCCCAAAAGCAGCTG; miR-223-5p: CGTGTATTTGACAAGCTGAGTT; miR-454-5p: ACCCTATCAATATTGTCTCTGC; let-7a-5p: ACGCTGAGGTAGTAGGTTG; let-7b-5p: GCTGAGGTAGTAGGTTGTG; and RNU6 (NR_002752.1): GCAAATTCGTGAAGCGTTCC [21,25,28,29,30,31,32,33]. Three cDNA samples were run in adjacent wells of each 96-well qPCR plate. The qPCR plates were run using the Real-Time PCR System (AriaMx, Agilent, Santa Clara, CA 95051, USA) using a two-step cycling protocol (95 °C for 5 min followed by 40 cycles of 95 °C for 5 s and 60 °C for 30 s), concluding with a melting curve (the dissociation temperature range extended from 60 °C to 90 °C). After the reactions, Ct values were determined using fixed-threshold settings (Ct thresholds: RNU6 = 2.3; miR-17-5p = 2.5; miR-24 = 2.2; miR-29b = 1.9; miR-30a-5p = 1.8; miR-146a = 1.7; miR-150-5p = 2.5; miR-155-5p = 2.3; miR-16-5p = 1.8; miR-191-5p = 2.2; miR-223-5p = 1.3; miR-454-5p = 1.9; let-7a-5p = 2.1; let-7b-5p = 1.8). The delta–delta Ct method and the 2^−ΔΔCT^ method were used to determine the relative amounts of miRNAs. The control sample included a minus reverse transcriptase (-RT) control and a no-template control (NTC). RNU6 was used as a reference gene to determine the relative expressions of the miRNAs.

### 2.8. Statistical Analysis

To compare the statistical differences between the term and premature groups, the non-parametric Mann–Whitney U test was used, using the Stata14 version 12.0 program for Windows. A *p*-value threshold of 0.05 was regarded as the level for statistical significance.

## 3. Results

The miRNA expressions were analyzed in human milk from mothers of preterm infants born between 26 and 36 weeks of gestation and from mothers of term infants within the first 48 h and at 30 days after birth. The maternal age and gender distributions did not differ between the two groups. Higher levels of fat and lactose were observed in the preterm human milk. No significant difference was observed in the protein concentrations (Table 1).

**Figure 1:**
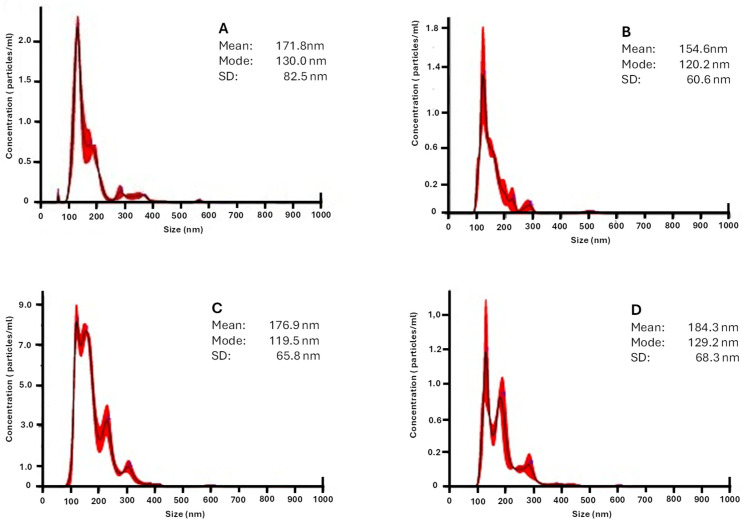
Exosome profiles at different temperatures. Profiles of the microvesicles obtained with fresh breast milk stored at −80 °C (**A**), fresh breast milk stored at −20 °C (**B**), old milk stored at −80 °C (**C**), and fresh milk preserved at 4 °C (**D**).

The protocols for the extraction of exosomes from breast milk, the storage conditions, and both the exosomes (NanoSight LM10 system, Malvern Panalytical, 7602 EA Almelo, The Netherlands) and extracted RNA (Spectrophotometer, Agilent, Santa Clara, CA 95051, USA) under different conditions have been previously standardized. Figure 1 shows the profiles of the microvesicles obtained with fresh breast milk stored at −80 °C (Figure 1A), fresh breast milk stored at −20 °C (Figure 1B), old fresh milk stored at −80 °C (Figure 1C), and fresh milk preserved at 4 °C (Figure 1D). The milk was immediately stored and extracted for one month at the temperatures described (Figure 1A,B,D). Sample C was kept for one year at −80 °C. The purified RNAs corresponded to the following concentrations. A: 305 ng/μL; B: 235 ng/μL; C: 214 ng/μL; and D: 200 ng/μL.

Based on our previous unpublished study, in which we analyzed the expression profiles of several miRNAs involved in the immune response in different mammalian milk sources, we determined the expression profiles of 13 miRNAs expressed in human milk.

We compared the expressions of miR-17-5p, miR-24, miR-29b, miR-30a-5p, miR-146a, miR-150-5p, miR-155-5p, miR-16-5p, miR-191-5p, miR-223-5p, miR-454-5p, let-7a-5p, and let-7b-5p in the skim and fat fractions of human milk from mothers of preterm and term infants.

The total concentration of RNAs was consistently higher in the milk fat fraction; therefore, all the following analyses were performed from the fat fractions of HM. The expressions of the miRNAs were analyzed at three different times of the day: morning, noon, and nighttime.

**Figure 2:**
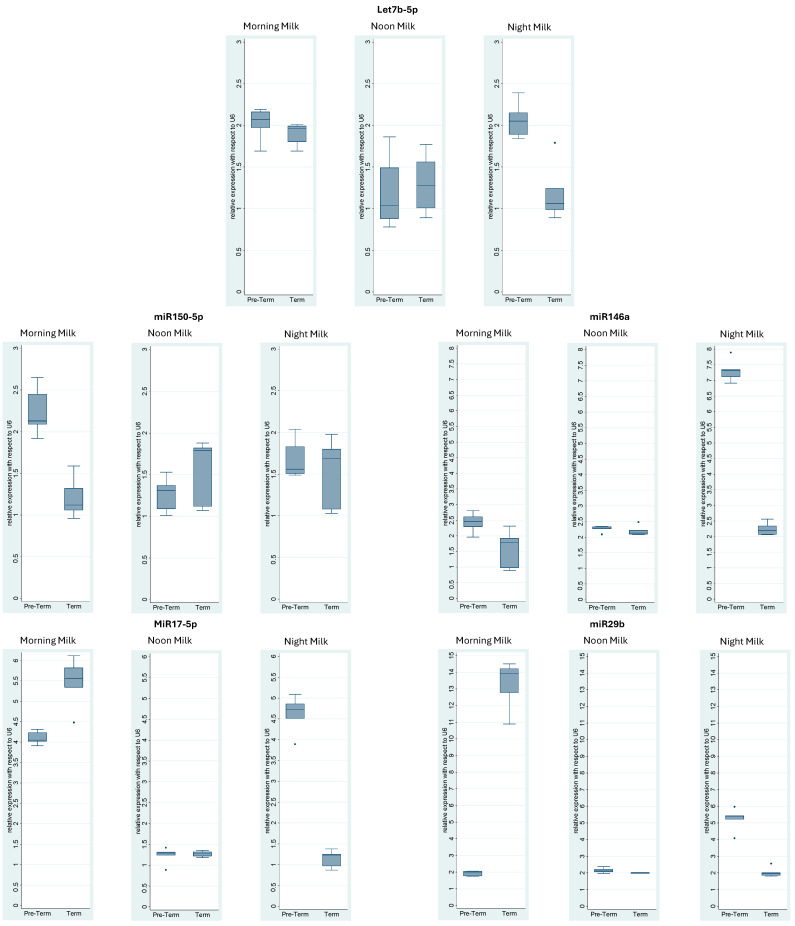

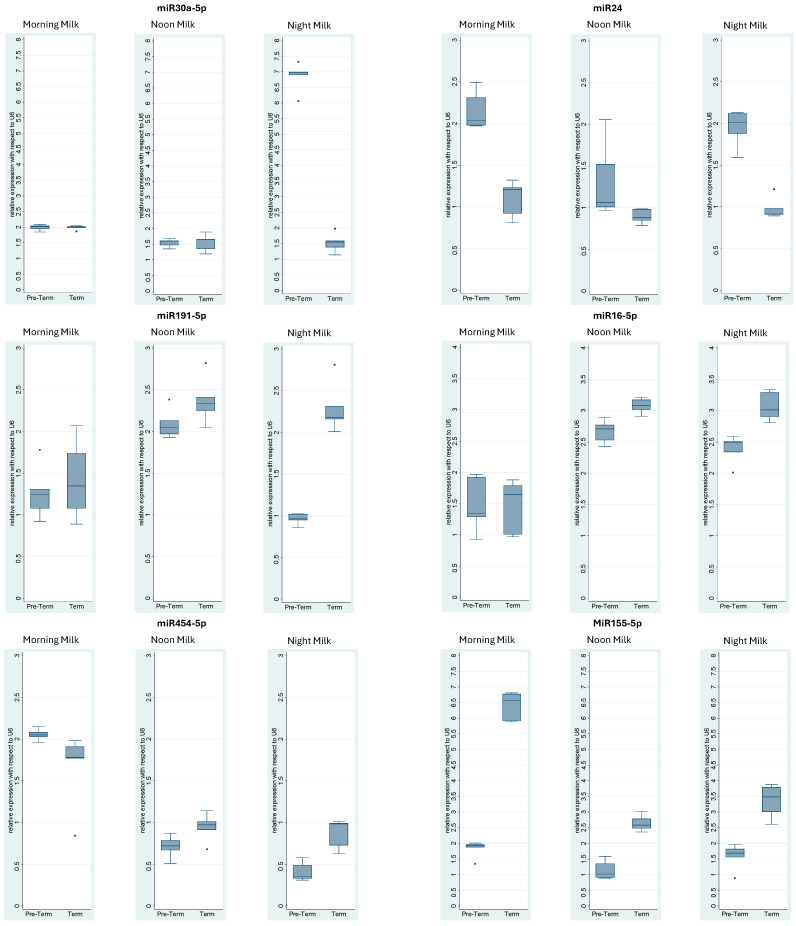

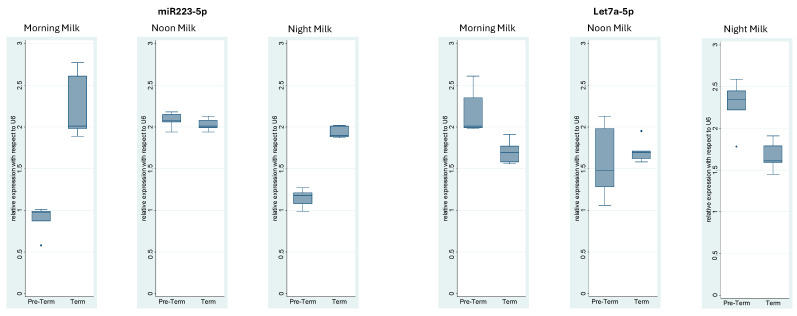
Boxplots of fold change values of miRNAs expressed in human milk. The boxplots represent median and interquartile ranges (IQRs) in the boxes and values for individual samples in the dots. Significant differentially expressed miRNAs were identified by filtering the results with a cut-off parameter-adjusted *p*-value < 0.05. *X*-axis shows preterm or term human milk. *Y*-axis represents fold change value of each transcript.

Figure 2 shows the miRNA expression levels from milk samples collected at morning, noon, and nighttime. The expression levels in the preterm and term human milk are compared. Table 2 summarizes the statistical data shown in Figure 2.

MiR-29b, involved in the innate immune response, was the most highly expressed in the HM. Its expression was significantly higher in morning milk compared with that in noon and nighttime milk (*p*-value < 0.009). The expression of miRNA-29b in the term HM was approximately 15-fold that in the premature HM at morning. At nighttime, however, the miRNA-29b expression in the preterm HM was approximately 5-fold that in the term HM (*p*-value < 0.009). No significant difference was observed at noon time (*p*-value < 0.0723) (Figure 2, Table 2). MiR-155-5p was the most highly expressed in the term HM at morning, noon, and nighttime versus its expression in the preterm HM (*p*-value < 0.009) (Figure 2, Table 2). The expression of miR-30a-5p in the preterm HM was approximately 7-fold that in the term human milk at nighttime (*p*-value < 0.009), whereas no significant difference was observed at morning or noon time (*p*-value < 0.0723) (Figure 2, Table 2). The expression of miR-146a was significantly higher in the preterm HM at morning and nighttime by two-folds in the morning (*p*-value < 0.0283) and seven-folds at nighttime (*p*-value < 0.009). However, no significant difference was observed at noon. The expression of miR-17-5p in the preterm HM was approximately 5-fold that in the term HM at nighttime. Nevertheless, its expression in the term HM was approximately 1.5-fold that in the preterm HM at morning (*p*-value < 0.009). No significant difference was observed at noon time. The expression of let-7b-5p was significantly higher in the preterm HM compared with that in the term HM at nighttime. The expression of miR-191-5p was significantly higher in the term HM than that in the preterm HM at night. No significant difference was observed at morning or at noon. MiR-24 was highly expressed in the preterm HM compared with that in the term HM at morning, noon, and nighttime (*p*-value < 0.0283). MiR-454-5p showed the high expression in the term HM at noon and nighttime compared with that in the preterm HM. At morning, the mir-454-5p expression was higher in the preterm HM that that in the term HM. The expression of miR-223-5p was higher in the term HM at morning and nighttime compared with that in the preterm HM. No significant difference was observed at noon time. The expression of let7a-5p was significantly higher in the preterm HM at morning and nighttime compared with that in the term HM. No significant difference was observed at noon time. MiR-16-5p showed higher expression in the term HM at noon and nighttime than in the preterm HM. No significant difference was observed at morning.

Finally, the expression of miR-150-5p was significantly higher in the preterm HM in the morning compared with that in the term HM. No significant difference was observed at noon or nighttime.

## 4. Discussion

Human breast milk is the most complete food that an infant can consume. It provides nutrients and many bioactive components that promote infant development [2]. Previous studies have shown how the contents of total fat, protein, and other nutrients exhibit circadian regulation in the quantity and quality of breast milk components [4]. Comparing the macromolecular contents of breast milk from mothers of preterm and term infants has also revealed differences in the contents of these nutrients. This indicates that the concentrations of nutrients and bioactive components in human breast milk adapt to the physiological needs of the infant [2,3,4].

Few studies have analyzed the expressions of miRNAs in breast milk comparing their profiles and concentrations in the milk of mothers of preterm and term infants [23,24]. The present study determined the expression levels of 13 miRNAs involved in immune signaling that are present in the human breast milk of mothers of preterm and term infants.

Macromolecule content in human breast milk. The significant increases in the fat and lactose contents in the milk of mothers of preterm infants coincide with previous studies that suggest a metabolic adaptation to meet the increased energy needs of these infants [19,34,35]. This result reflects the biological adaptation aimed at meeting the accelerated energy and metabolic needs of these infants [36]. Various meta-analyses indicate that the protein concentrations are higher in the milk of mothers of preterm infants than those in the milk of mothers of term infants [36,37]. However, in our study, the protein concentrations did not show significant differences, suggesting a more stable regulation of this nutritional component. Although the concentration of total proteins shows no differences, we cannot rule out the existence of different proportions of proteins in the breast milk. The limited number of samples studied does not permit further analysis of this data. Further analysis would allow us to determine the amounts of different proteins in breast milk.

Expressions of immunoregulatory miRNAs. The results show the increased expressions of miR-17-5p, miR-24, miR-29b, miR-30a-5p, and miR-146a in the night milk of mothers of preterm infants, revealing a relevant circadian pattern. This supports the idea that human breast milk varies not only in its nutritional composition but also in its immunomodulatory potential throughout the day, possibly adapting to the rhythms of the infant’s physiological needs. MiR-17-5p, miR-24, miR-29b, miR-30a-5p, and miR-146a are involved in the regulation of innate immune responses and inflammatory processes. Their overexpression at night suggests a mechanism of immunological compensation [13,18].

The circadian miRNA expression has been widely described in the literature. microRNAs such as miR181, miR191, miR192, miR122, miR125, miR279, and miR132 show differential circadian expressions [38,39,40,41,42,43]. However, there is poor scientific evidence of the miRNA expression in HM showing circadian rhythm. Our results are consistent with the differential expressions of miRNAs in breast milk following circadian cycles, such as miR-16-5p [44,45,46,47]. The observed diurnal variability in the expressions of these miRNAs, particularly their higher levels at night, suggests that human breast milk has a functional circadian rhythm, which may influence the development of the infant’s immune system [48]. miR16-5p targets are involved in reducing cell proliferation directed to the 3′UTRs of G1/S regulatory genes. miR16-5p targets in Cyclin D1 (Ccnd1), cyclin D2 (Ccnd2), cyclin D3 (Ccnd3), cyclin E (Ccne1), and cyclin-dependent kinase 6 (Cdk6) [45]. These results are according to the dynamic regulation of the bioactive components in human breast milk related to the maternal and infant physiological conditions [16]. The expressions of miR-17-5p, miR-24, and miR-146a show statistically significant differences between groups and times of day, especially in nighttime milk.

Given that they are involved in inflammatory processes and the regulation of innate immunity [48,49], their increased presence in the milk of mothers of preterm infants could represent an immunological compensation mechanism for infants with less developed immune systems. Regarding the expression levels of miR-29b and miR-30a-5p, variable levels of these transcripts were observed, which were particularly elevated at night in milk samples from mothers of preterm infants. These miRNAs have been implicated in the signaling pathways of immunological maturation and tissue development [50,51]. Overexpression at specific times may be synchronized with moments of the greatest physiological receptivity in the newborn. The expressions of miR-16-5p and miR-191-5p did not show statistically significant differences. These results may be related to a function less dependent on the infant’s maturity status or the less direct immunological relevance in this context, as Godoy et al. have suggested [52]. MicroRNAs such as miR-150-5p, miR-155-5p, and miR-454-5p showed specific differences in their expressions. However, no circadian patterns or differences between the preterm and term groups were identified. These miRNAs participate in the immune response regulation pathway, fulfilling more specific functions [53,54]. The expressions of these miRNAs could be modulated by other factors, such as maternal diet, health status, or genetic makeup [55,56].

Finally, the findings of this study reinforce the complex and dynamic role of human breast milk as a vehicle not only for essential nutrients but also for functional genetic information in the form of microRNAs (miRNAs). As described by de la Torre Gomez et al. [3], exosomes present in human milk contain miRNAs capable of modulating gene expression in infants, particularly in immunological processes. In our sample, marked differences in the expressions of certain miRNAs were observed between the milk of mothers of preterm and term infants, especially at night [17,57,58].

Some limitations of this study include the following. The study was preliminary and based on a small sample (*n* = 10), which limits the generalizability of the results. Variables such as maternal diet or emotional status, which could influence the milk composition, were not controlled for. Although the exclusion criteria were defined to exclude mothers with known pathologies or infants whose growth was not commensurate with their age, it is possible that many other factors modified the breast milk composition. The analysis was restricted to only 13 miRNAs, without including functional studies confirming their immunological impact. Although diurnal variations in the expressions of some miRNAs were observed, their clinical relevance was not assessed. Future larger longitudinal studies are needed to validate these findings.

## 5. Conclusions

This preliminary study reveals that human breast milk from mothers of preterm infants exhibits greater variability and higher levels of expression of certain immunomodulatory miRNAs, especially at night.

As a brief preliminary study, there are several factors that regulate the composition of human breast milk which were not analyzed. Therefore, the differences found may not only be due to the infant’s condition but also to aspects of the mother’s lifestyle.

Nevertheless, these findings reinforce the hypothesis that human breast milk acts as a dynamic adaptive system that responds to the specific needs of the newborn. Human breast milk feeding promotes the development of the infant’s brain, gut, and immune system, among others, through molecular mechanisms involving regulatory pathways gated by TNF-α, IL-6, IFN-β, PD-L1, JAK1/JAK3, the Notch signaling pathway, and TLR7. Larger studies are needed to confirm these patterns and explore their long-term clinical impacts to direct miRNA–infant outcome correlations.

## Figures and Tables

**Table 1:** Clinical characteristics of mothers and infants and nutritional components.

	Term (5)	Preterm (5)
Characteristics of mothers and infants		
Maternal age (years)	33.01 ± 6.42	34.35 ± 2.84
Gestational age (week) *	39.62 ± 1.82	31.58 ± 1.92
Birth weight (grams) *	3445.65 ± 280.72	1748.45 ± 503
C-section (%)	40	80
Males (%)	20	80
Females (%)	80	20
Nutritional components		
Fat (%) *	0.86 ± 0.12	2.24 ± 0.51
Protein (%)	1.21 ± 0.15	1.33 ± 0.12
Lactose (%) *	4.48 ± 1.10	7.09 ± 0.48

* *p*-value < 0.05.

**Table 2:** Statistical analysis of microRNA expression values. Mean, standard deviation, and *p*-values are shown. * *p*-values < 0.05 were considered statistically significant.

ncRNA	Period	Mean	S. D.	*p*-Value
miR-17-5p	Morning milk preterm	4.104	0.163	0.0090 *
	Morning milk term	5.464	0.622	
	Noon milk preterm	1.230	0.20	1.0000
	Noon milk term	1.272	0.070	
	Night milk preterm	4.616	0.457	0.0090 *
	Night milk term	1.140	0.212	
miR-24	Morning milk preterm	2.158	0.231	0.0090 *
	Morning milk term	1.098	0.220	
	Noon milk preterm	1.324	0.467	0.0283 *
	Noon milk term	0.898	0.086	
	Night milk preterm	1.946	0.223	0.0088 *
	Night milk term	0.98	0.133	
miR-29b	Morning milk preterm	1.926	0.144	0.0090 *
	Morning milk term	13.26	1.477	
	Noon milk preterm	2.154	0.153	0.0723
	Noon milk term	2.004	0.023	
	Night milk preterm	5.216	0.696	0.0090 *
	Night milk term	2.046	0.297	
miR-30a-5p	Morning milk preterm	1.988	0.090	0.7526
	Morning milk term	1.980	0.066	
	Noon milk preterm	1.544	0.134	0.7540
	Noon milk term	1.546	0.274	
	Night milk preterm	6.850	0.468	0.0090 *
	Night milk term	1.530	0.307	
miR-146a	Morning milk preterm	2.424	0.323	0.0283 *
	Morning milk term	1.578	0.618	
	Noon milk preterm	2.266	0.103	0.2948
	Noon milk term	2.200	0.166	
	Night milk preterm	7.318	0.365	0.0090 *
	Night milk term	2.244	0.210	
miR-150-5p	Morning milk preterm	2.248	0.295	0.0090 *
	Morning milk term	1.210	0.250	
	Noon milk preterm	1.262	0.211	0.2506
	Noon milk term	1.536	0.404	
	Night milk preterm	1.686	0.240	0.6015
	Night milk term	1.516	0.434	
Mir-155-5p	Morning milk preterm	1.828	0.271	0.0090 *
	Morning milk term	6.392	0.453	
	Noon milk preterm	1.152	0.306	0.0090 *
	Noon milk term	2.644	0.261	
	Night milk preterm	1.586	0.418	0.0090 *
	Night milk term	3.352	0.535	
miR-16-5p	Morning milk preterm	1.49	0.445	0.7540
	Morning milk term	1.46	0.437	
	Noon milk preterm	2.666	0.186	0.0090 *
	Noon milk term	3.078	0.120	
	Night milk preterm	2.388	0.230	0.0090 *
	Night milk term	3.068	0.232	
miR-191-5p	Morning milk preterm	1.268	0.324	0.6752
	Morning milk term	1.426	0.481	
	Noon milk preterm	2.092	0.178	0.0937
	Noon milk term	2.372	0.284	
	Night milk preterm	0.966	0.068	0.0090 *
	Night milk term	2.294	0.307	
miR-223-5p	Morning milk preterm	0.884	0.178	0.0090 *
	Morning milk term	2.252	0.406	
	Noon milk preterm	2.083	0.093	0.3443
	Noon milk term	2.03	0.075	
	Night milk preterm	1.146	0.111	0.0090 *
	Night milk term	1.936	0.073	
miR-454-5p	Morning milk preterm	2.054	0.694	0.0163 *
	Morning milk term	1.656	0.464	
	Noon milk preterm	0.71	0.134	0.0472 *
	Noon milk term	0.942	0.169	
	Night milk preterm	0.412	0.118	0.0090 *
	Night milk term	0.868	0.176	
Let-7a-5p	Morning milk preterm	2.188	0.282	0.0090 *
	Morning milk term	1.702	0.144	
	Noon milk preterm	1.586	0.456	0.6015
	Noon milk term	1.710	0.144	
	Night milk preterm	2.278	0.310	0.0283 *
	Night milk term	1.670	0.181	
Let-7b-5p	Morning milk preterm	2.016	0.201	0.2087
	Morning milk term	1.890	0.139	
	Noon milk preterm	2.016	0.201	0.6015
	Noon milk term	1.890	0.139	
	Night milk preterm	2.064	0.220	0.0090
	Night milk term	1.194	0.357	

## Data Availability

The data presented in this study are available upon request from the corresponding author.
